# Prevalence of bacterial species associated with ovine footrot and contagious ovine digital dermatitis in Swedish slaughter lambs

**DOI:** 10.1186/s13028-022-00625-2

**Published:** 2022-03-09

**Authors:** Anna Rosander, Rebecka Albinsson, Ulrika König, Ann Nyman, Sara Frosth

**Affiliations:** 1 Department of Biomedical Sciences and Veterinary Public Health, Faculty of Veterinary Medicine and Animal Science, Swedish University of Agricultural Sciences (SLU), P.O. Box 7036, 750 07 Uppsala, Sweden; 2 District Veterinarians Tibro, Norra Vägen 9, 543 35 Tibro, Sweden; 3 Farm & Animal Health, Kungsängens Gård, 753 23 Uppsala, Sweden; 4 Department of Clinical Sciences, Faculty of Veterinary Medicine and Animal Science, Swedish University of Agricultural Sciences (SLU), P.O. Box 7054, 750 07 Uppsala, Sweden

**Keywords:** Abattoir, CODD, *Dichelobacter nodosus*, *Fusobacterium necrophorum*, Real-time PCR, Swab samples, *Treponema* species

## Abstract

**Background:**

Ovine footrot and contagious ovine digital dermatitis (CODD) are contagious mixed bacterial infections with major impacts on animal health and production. In Sweden, ovine footrot and CODD were first detected in 2004 and 2019, respectively. In 2009, a voluntary control programme for footrot was established, and a prevalence study in slaughter lambs was conducted, however, the distribution of footrot and CODD-associated bacteria is still unknown. This study examined the prevalence of *Dichelobacter nodosus, Fusobacterium necrophorum* and *Treponema* spp., as well as the current prevalence of footrot and CODD, in Swedish slaughter lambs.

**Results:**

A total of 2048 feet, from 512 slaughter lambs, were collected from eight slaughterhouses throughout Sweden in autumn 2020. All feet were visually examined for lesions of footrot and CODD and sampled for subsequent real-time polymerase chain reaction (PCR) analysis. Nine lambs (1.8%) had at least one foot affected with footrot (footrot score ≥ 2). A CODD grade 1 lesion was detected in a single lamb (0.2%). The prevalence of *D. nodosus, F. necrophorum* and *Treponema* spp. was 6.1%, 7.6% and 90.6%, respectively. The *D. nodosus* detected were benign strains.

**Conclusions:**

The prevalence of footrot in Swedish slaughter lambs has been significantly reduced, from 5.8 to 1.8%, during the past 11 years. This indicates that preventive measures, such as the national control programme and elimination of footrot from affected flocks, have been effective. A single lamb (0.2%) was found with a CODD lesion (grade 1). In Sweden, benign rather than virulent strains of *D. nodosus* seem to be the most common. Neither *D. nodosus* nor *F. necrophorum* were widespread among Swedish slaughter lambs, but both were more likely to be found in lambs with footrot. *Treponema* spp. was very commonly found in lambs with and without footrot, but there is a lack of information on the individual *Treponema* spp. present in Swedish slaughter lambs and their potential pathogenicity.

## Background

Ovine footrot is a contagious disease that causes pain and lameness in affected animals [1]. The causative bacterium is believed to be the anaerobic and Gram-negative *Dichelobacter nodosus*, but other bacteria such as *Fusobacterium necrophorum* and *Treponema* spp. may be involved in the development and progression of the disease [1–7]. Footrot lesions range from mild inflammation of the interdigital skin (benign footrot) to complete underrunning of the hoof capsule (virulent footrot) [1, 8]. Disease severity depends on the virulence of the *D. nodosus* strain causing the infection [9], host susceptibility [10] and environmental factors [11, 12]. *D. nodosus* strains have traditionally been referred to as virulent or benign based on the severity of disease caused under ideal climatic conditions [13]. More recently, whole-genome sequencing has shown that virulent and benign strains differ in one of the acidic proteases (AprV2/B2) responsible for virulence [14], and real-time polymerase chain reaction (PCR) assays have been developed to differentiate between the two variants [15, 16].

Footrot in sheep occurs world-wide and was first reported in Sweden in 2004 [17]. In 2009, a footrot prevalence study was conducted in slaughter lambs and found to be 5.8% at an individual animal level [18]. In Sweden, footrot is defined as the presence of at least one foot with footrot score ≥ 2 according to the scoring system devised by Stewart and Claxton [8] (Table 1). When the term footrot is used here, it is the Swedish definition that is referred to unless specifically stated otherwise. Footrot score 1 is not considered to represent footrot disease in Sweden, as a footrot score 1 can also be due to environmental factors. However, the study in 2009 [18] was based solely on pathological findings, and not detection of *D.* *nodosus*, and hence the distribution of the bacterium in Sweden is still unknown. Consequently, the proportion of asymptomatic carriers is also unknown, which has important implications for the control of the disease. It has previously been reported that benign as well as virulent strains of *D. nodosus* can be present in healthy sheep [12, 19–22], but this has not yet been confirmed for a random sample set in Sweden. Furthermore, the prevalence of *F. necrophorum* and *Treponema* spp. in Swedish sheep has yet to be investigated.

**Table 1:** Footrot scoring system from Stewart and Claxton [8] and the Swedish definition of footrot used in this study

Footrot score	Description	Swedish definition
0	Healthy foot	Not footrot
1	Inflammation of the interdigital skin with erosion of the epithelium	Not footrot
2	Necrotising inflammation of the interdigital skin and part or all of the soft horn of the axial wall of the digit	Footrot
3	Necrotising inflammation and underrunning of part or all of the soft horn of the heel and sole, not extending to the abaxial edge of the sole	Footrot
4	Underrunning extending to the abaxial edge of the sole	Footrot
5	Necrotising inflammation of the laminae of the abaxial wall and underrunning of the hard horn	Footrot

*D.* *nodosus, F. necrophorum* and *Treponema* spp. have previously been found in contagious ovine digital dermatitis (CODD) lesions [23]. CODD causes pain and lameness in affected animals and early cases typically present with erosion/ulceration at the coronary band. The two outbreaks of CODD that have occurred in Sweden were diagnosed in 2019 and 2020 [24], and involved several animals on two farms, both located in southern Sweden [24]. Footrot and CODD have recently been suggested to be different stages of the same disease, rather than different diseases [25], and five pathogens (*D. nodosus, F. necrophorum, Treponema medium, T. phagedenis* and *T. pedis*) have been found to be associated with all stages of these foot diseases [26].

Footrot has been a notifiable disease in Sweden since 2008 and initially all sheep flocks testing positive for *D. nodosus* had to be reported to the Swedish Board of Agriculture. However, in 2021, the notification obligation changed to include only virulent strains of *D. nodosus*. A voluntary control programme for footrot was established in Sweden in 2009; by 2020, 328 out of 7900 Swedish sheep flocks were affiliated with the programme [27]. The programme aims to eliminate footrot, facilitate trade of footrot-free animals through a certification system, and to train veterinarians, sheep farmers and non-veterinary staff to perform clinical inspections and footrot scoring [27].

In this study, collection of lamb feet at slaughterhouses was used as the sampling strategy, though it should be noted that lambs with more severe foot lesions are not normally sent for slaughter, since they often are lame and under antibiotic treatment. Thus this strategy could lead to an underestimation of footrot prevalence, however, it was selected because it allowed a larger number of animals to be sampled in an efficient way. In addition, the procedure is non-invasive and ensured that the samples were relatively representative of the country, since the vast majority of sheep flocks send lambs for slaughter. This sampling strategy has also been used by others for surveillance of footrot [28], including the previous footrot prevalence study in Sweden [18], making the results comparable over time.

The aims of this study were to determine the prevalence of *D. nodosus, F. necrophorum* and *Treponema* spp. in Swedish slaughter lambs and to determine the current prevalence of footrot and CODD.

## Methods

### Selection of slaughterhouses and collection of lamb feet

**Fig. 1:**
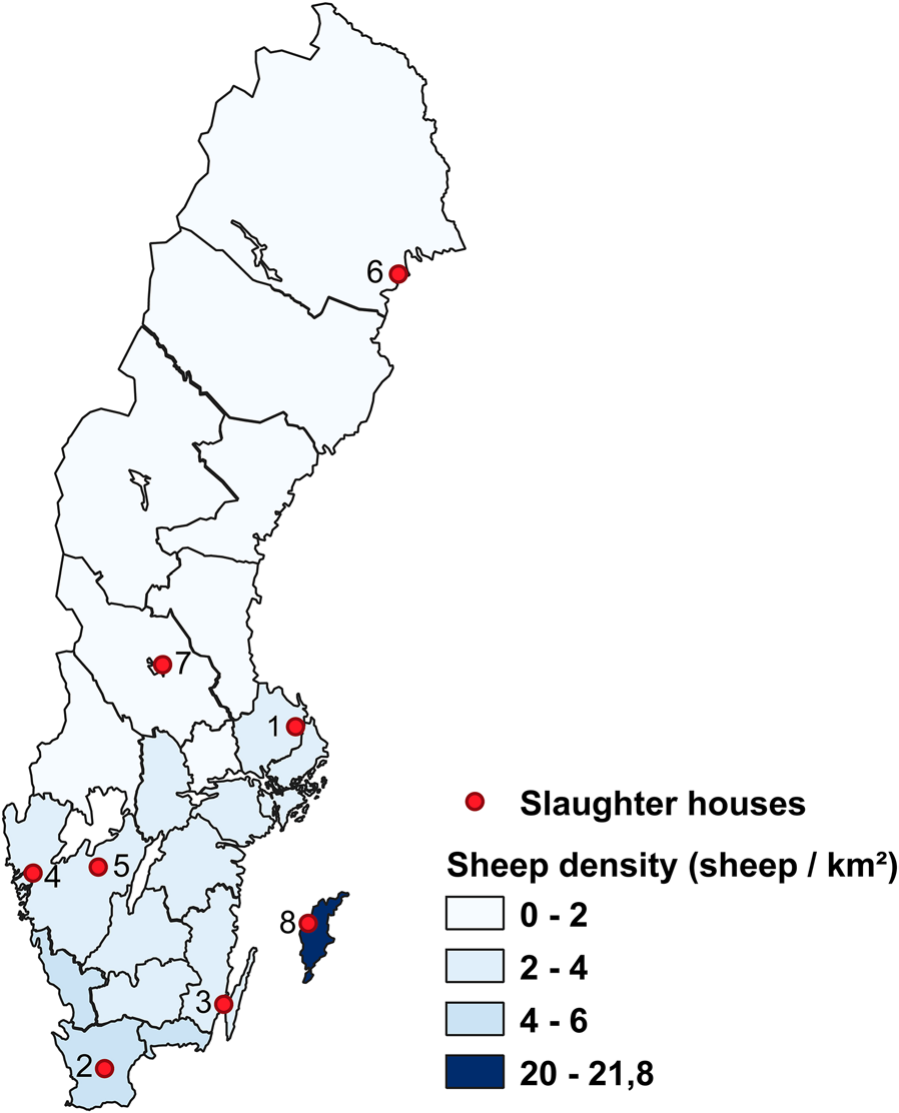
County-level sheep density in Sweden in 2019 and geographical location (1–8) of the eight selected slaughterhouses. **1** Alunda; **2** Hörby; **3** Kalmar; **4** Ljungskile; **5** Skara; **6** Luleå; **7** Vikarbyn; **8** Visby. Locations 1–5 are in southern Sweden, locations 6–7 in northern Sweden and location 8 on Gotland. Diagram created in QGIS 3.20.1-Odense (Free Software Foundation Inc., Boston, MA, USA) using sheep data from Swedish agricultural statistics compiled in 2020 [30] and map and area data from Statistics Sweden (https://scb.se/)

Slaughterhouses were selected based on the number of lambs slaughtered in 2019 as well as geographical location, with the aim to include the highest-volume slaughterhouses and to achieve a distribution representative of slaughter lambs across Sweden. Eight slaughterhouses were selected, with the majority located in southern Sweden, including the island of Gotland, which has the highest sheep density in the country (Fig. 1).

The number (n) of lambs to be sampled was calculated using the equation [29]:

$${\text{n = }}\left( {{\text{Z}}^{{2}} \times {\text{P }}\left( {1 - {\text{P}}} \right)} \right)/{\text{d}}^{2}$$

where Z is the z-score associated with a level of confidence, P is prevalence and d represents precision. The prevalence was found to be 5.8% for footrot in 2009 [18], but no prevalence value was available for CODD and hence it was set to 50%. The precision (d) was set to 5% and using a 95% level of confidence gave Z = 1.96, resulting in n = approximately 400 lambs. However, the number was adjusted upwards to 512 (2048 lamb feet) to compensate for diagnostic sensitivity and possible loss of samples. A total of 500 lambs were included in the previous study in Sweden [18]. The number of lambs sampled at each slaughterhouse was set proportional to its slaughter volume during 2019. The total number of slaughtered lambs in Sweden during 2019 was 213,601 [30].

Collection of lamb feet took place during September–October 2020 and was performed by slaughterhouse staff. Autumn was chosen as the study period since it follows the summer, which in Sweden has relatively warm and moist weather considered to favour the clinical manifestation of footrot. Autumn is also the period when the most lambs are slaughtered in Sweden. Staff were given detailed instructions on how to collect the feet and, in order to include as many flocks as possible, collection was performed for at least every tenth slaughtered lamb. From the selected lambs, all four feet were cut off, placed in plastic bags and sent by regular mail in padded envelopes, without special cooling, to the Department of Biomedical Sciences and Veterinary Public Health at the Swedish University of Agricultural Sciences (SLU). The majority of feet were dispatched directly after sampling and arrived at the laboratory within 1–3 days, with the exception of feet from Gotland, which for practical reasons were kept in cold storage for up to one week before being sent by regular mail.

The study was conducted in accordance with Swedish animal health regulations. Sampling of the animals was performed after slaughter and thus no ethical permission was required. SLU has a general permit to use animal by-products (Swedish Board of Agriculture, diary no. 6.7.18-13720/2019).

### Pathological assessment of lamb feet

All 2048 feet were visually examined by at least two individuals trained by the Farm & Animal Health veterinarian, who is responsible for the national footrot control programme. The feet were not cleaned prior to sampling to prevent bacteria from being washed away, with the exception of manual removal of large pieces of manure or straw. The 1–5 scoring system developed by Stewart and Claxton [8] (Table 1) was used to assess the feet for footrot lesions, while the 1–5 grading system developed by Angell et al. [31] (Table 2) was used for CODD scoring. The lambs were given an overall lesion score according to the highest score of any one foot. For a lamb to be categorised as affected by footrot, at least one foot with footrot score ≥ 2 was required. However, footrot score 1 lesions were recorded separately from footrot score 0, in contrast to previous studies, where no distinction was made between these scores [16, 18].

**Table 2:** CODD grading system from Angell et al. [31] used in this study

CODD grade	Description
0	Healthy foot
1	Focal lesion, either proliferative or erosive/ulcerative, affecting the digital skin and coronary band. No underrunning of the hoof capsule
2	Separation between the hoof capsule and the lamellae affecting < 50% of the hoof wall. Swollen digits. Subcorneal tissue haemorrhagic often with purulent material adherent. Foul smell
3	As grade 2 but affecting 50–100% of the hoof wall
4	Horn beginning to regrow. Exposed subcorneal tissue roughened, haemorrhagic/necrotic, friable and easily traumatised. Affected digits often distinctly swollen and shortened
5	Horn regrown, surface smooth but distorted by circumferential ridges or creases. Digit often still wide and short

### Sampling and bacterial DNA extraction

Sampling was performed by the same persons who scored the feet, and was done with great care to ensure consistency between the samples. For example, one person held the feet in the correct position while another person performed the sampling by spinning the swab one turn. The interdigital skin of all 2048 lamb feet was sampled with a FLOQSwab (Copan Italia s.p.a., Brescia, Italy) and the swabs were pooled by individual lamb into 50 mL centrifuge tubes containing 4 mL liquid transport medium. The transport medium used was prepared by the National Veterinary Institute (SVA; Uppsala, Sweden) using the recipe in Amies et al. [32], with the exception that agar and charcoal were excluded. The centrifuge tubes were shaken for 5 min at 700–800 rpm and 2 mL of the liquid was transferred to a 2 mL microcentrifuge tube. Pre-treatment of the samples and DNA extraction were then performed as previously described [33]. In short, the samples were pelleted and lysed by G2 buffer (Qiagen, Hilden, Germany) and proteinase K (Qiagen). The extraction was performed using the EZ1 DNA Tissue Kit (Qiagen) and the EZ1 DNA Bacterial protocol, in combination with the EZ1 Advanced XL instrument (Qiagen).

In addition, CODD lesions were sampled using an ESwab (Copan Italia s.p.a.) at the coronary band. DNA extraction was performed in the same way as for the pooled interdigital skin samples, but the volume used for DNA extraction was 0.5 mL liquid Amies transport medium, which was approximately half the volume of liquid in the ESwab.

### Real-time PCR

The DNA extracted from all 512 pooled samples and the CODD samples were subjected to real-time PCR analysis for the presence of *D. nodosus* as previously described [16]. In addition, *D. nodosus*-positive samples were analysed by the *aprV2/B2* assay developed by Frosth et al. [16] for virulence determination. All DNA extracts were also analysed for the presence of *F. necrophorum* and *Treponema* spp. by real-time PCR methods described in Jensen et al. [34] and Strub et al. [35], respectively, with some modifications. Both PCR assays were run using the TaqMan Fast Advanced Master Mix (Thermo Fisher Scientific Inc., Waltham, MA, USA) with a total mastermix volume of 15 µL. Bovine serum albumin (BSA) (Sigma-Aldrich, St Louis, MO, USA) was included at a final concentration of 0.1 mg/mL and the TaqMan Exogenous Internal Positive Control Reagents (Thermo Fisher Scientific Inc.) were included as controls for possible PCR inhibition. The PCR programme consisted of 10 min at 95 °C, followed by 45 cycles of 95 °C for 30 s and 60 °C for 1 min. In addition, the *Fusobacterium* assay was run with the two subspecies-specific probes in duplex, and not separately.

All PCR assays were run on a CFX Opus 96 Real-Time PCR Instrument (Bio-Rad Laboratories Inc., Hercules, CA, USA) and analysed with the CFX Maestro Software version 2.0 (Bio-Rad Laboratories Inc.) with default settings. Samples were considered positive if they generated probe-specific fluorescent signals of quantification cycle (Cq) < 40. All PCR runs contained at least one negative control consisting of DNase- and RNase-free water (Sigma-Aldrich) and all PCR runs included positive controls. For *D. nodosus,* isolates AN363/05 *(aprV2* positive) and AN484/05 *(aprB2* positive) (SVA) were used. The *F. necrophorum* subspecies *necrophorum* CCUG 9994T and *F. necrophorum* subspecies *funduliforme* CCUG 42162T type strains (Culture Collection, University of Gothenburg, Sweden) were used in the *Fusobacterium* assay. The *T. pedis* DSM 18691 type strain (Leibniz Institute DSMZ-German Collection of Microorganisms and Cell Cultures GmbH, Braunschweig, Germany) was used as a positive control in the *Treponema* spp. assay.

### Statistical analysis

The distributions of findings were summarised in Excel, while all statistical analyses were performed in Stata 17.0 (StataCorp. 2021. College Station, TX: StataCorp LLC). The difference in prevalence of lambs with footrot between the present and the previous (2009) national screening was assessed using a two-sample test of proportions (using the prtest command in Stata 17.0). Associations between footrot scorings (dependent variable), detections of *D. nodosus*, *F. necrophorum*, *Treponema* spp., individually as well as in combination, and region (independent variables) were investigated using univariable multinomial logistic regression analysis (using the mlogit command in Stata 17.0). To investigate the differences between all categories of the dependent variable, this model was re-run changing the base category until all categories had been compared with each other. Associations between detections of *D. nodosus*, *F. necrophorum* and *Treponema* spp. (all as dependent variables in separate analyses) and region (independent variable) were investigated by univariable logistic regression analysis (using the logit command in Stata 17.0). As only univariable analyses were performed, the fit of the multinomial logistic and the logistic models was not investigated.

## Results

### Prevalence of footrot and CODD

**Fig. 2:**
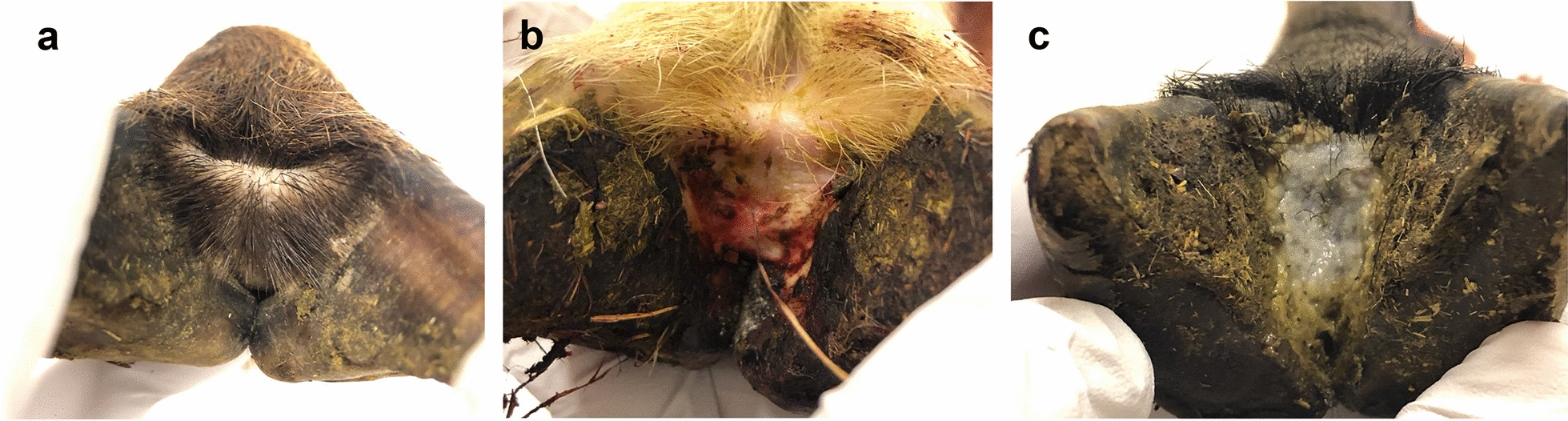
Typical appearance of lamb feet with footrot score 0 (**a**), 1 (**b**) and 2 (**c**) found in this study. **a** Healthy foot with dry interdigital skin covered with a normal amount of hair; **b** foot with footrot score 1 with inflammation of the interdigital skin which can be seen through hair loss and redness of the skin; **c** foot with footrot score 2 with necrotising inflammation of the interdigital skin and skin horn junction covered with grey exudate and foul smell

Of the 2048 lamb feet investigated, 17 were categorised as footrot score 2, 140 as footrot score 1 and 1891 as footrot score 0 (Table 3, Fig. 2). No feet with footrot score ≥ 3 were detected in the study. On an individual level, 1.8% of the lambs had at least one foot with footrot score 2, 14.6% had footrot score 1 and 83.6% had footrot score 0 (Table 3). Thus, 1.8% of the lambs in the study had footrot (Table 3). The prevalence was significantly lower than the level (5.8%) found in 2009 (P < 0.001) [18].

**Table 3:** Distribution of *Dichelobacter nodosus*, *Fusobacterium necrophorum* and *Treponema* spp. and footrot scores in Swedish slaughter lambs

	Footrot score 0 (%)	Footrot score 1 (%)	Footrot score 2 (%)	Total
Number of feet studied	1891 (92.3)	140 (6.8)	17 (0.8)	2048
Number of lambs represented	428 (83.6)	75 (14.6)	9 (1.8)	512
Number of lambs with *D. nodosus*	14 (3.3)	10 (13.3)	7 (77.8)	31
Number of lambs with *F. necrophorum*	29 (6.8)	6 (8.0)	4 (44.4)	39*
Number of lambs with *Treponema* spp.	384 (89.7)	71 (94.7)	9 (100)	464

*In two samples (both footrot score 2), *F. necrophorum* subsp. *necrophorum* was detected. In the remaining 37 samples, *F. necrophorum* subsp. *funduliforme* was detected.

Only one foot from a single lamb had a CODD lesion (grade 1) in this study (0.2%), but 39 (7.6%) lambs had a superficial lesion or hair loss in the coronary band in at least one foot. All feet of the lamb with CODD grade 1 were scored as footrot score 0.

### Real-time PCR of pooled swab samples

#### *Dichelobacter nodosus*

A total of 31 (6.1%; 95% CI 4.2–8.5%) of the 512 lambs tested positive for *D. nodosus* by real-time PCR. The proportion of *D. nodosus* findings was highest in the nine lambs with footrot score 2 (77.8%), but the bacterium was also detected in lambs with footrot score 1 (13.3%) and 0 (3.3%) (Table 3). The results of the regression analysis showed a significantly higher relative risk ratio (RRR) of finding *D. nodosus* in lambs with footrot score 2 than in lambs with footrot score 1 (RRR = 22.7; 95% CI 4.13–125.37; P < 0.001) or score 0 (RRR = 103.50; 95% CI 19.69–543.94; P < 0.001). The RRR of finding *D. nodosus* was also significantly higher in lambs with footrot score 1 than in lambs with footrot score 0 (RRR = 4.55; 95% CI 1.94–10.67; P < 0.001).

Twenty-six (83.9%) of the 31 *D. nodosus*-positive lambs tested positive for the *aprB2* gene in the virulence PCR, and the *D. nodosus* involved was therefore considered benign. No lamb tested positive for the *aprV2* gene, and hence no virulent *D. nodosus* was detected in this study. The *D. nodosus* from five lambs, two with footrot score 1 and three with score 0, tested negative for both genes.

#### *Fusobacterium necrophorum*

A total of 39 (7.6%; 95% CI 5.5–10.3%) of the 512 lambs tested positive for *F. necrophorum* by real-time PCR. As seen for *D. nodosus*, the proportion of *F. necrophorum* was highest in lambs with footrot score 2 (44.4%), but it was also present in lambs with footrot score 1 (8.0%) and 0 (6.8%) (Table 3). The RRR of finding *F. necrophorum* was significantly higher in lambs with footrot score 2 than in lambs with footrot score 1 (RRR = 9.20; 95% CI 1.94–43.65; P = 0.005) and 0 (RRR = 11.01; 95% CI 2.80–43.22; P = 0.001), but not in lambs with footrot score 1 compared with lambs with footrot score 0 (P = 0.70). The real-time PCR for *F. necrophorum* distinguished between the two subspecies *necrophorum* and *funduliforme*. Two samples (5.1%) tested positive for *F. necrophorum* subsp. *necrophorum*, both from lambs with footrot score 2, while the remaining 37 samples (94.9%) tested positive for *F. necrophorum* subsp. *funduliforme*. The RRR of finding *F. necrophorum* subsp. *necrophorum* was significantly higher in lambs with footrot score 2 than in lambs with footrot score 1 (RRR = 64.25; 95% CI 63.48–65.02; P < 0.001) and 0 (RRR = 64.25; 95% CI 63.50–65.00; P < 0.001). There was no significant difference in RRR of finding *F. necrophorum* subsp. *necrophorum* between lambs with footrot score 1 or 0 (P = 1.00). There was no significant difference in RRR between footrot score and findings of *F. necrophorum* subsp. *funduliforme* (P = 0.33).

#### *Treponema* spp.

A total of 464 (90.6%; 95% CI 87.8–93.0%) of the 512 lambs tested positive for *Treponema* spp. by real-time PCR. *Treponema* spp. was detected in 89.7%, 94.7% and 100% of the samples from lambs with footrot score 0, 1 and 2, respectively (Table 3). There was no significant association between footrot score and the presence of *Treponema* spp. (P = 0.14).

#### Combined analysis of *Dichelobacter nodosus, Fusobacterium necrophorum* and *Treponema* spp.

When combining all the bacterial results, 9.2% of the 512 lambs had no bacterial finding, 0.2% had findings of only *F. necrophorum*, 78.2% had findings of only *Treponema* spp., 4.7% had findings of both *D. nodosus* and *F. necrophorum*, 6.1% had findings of both *F. necrophorum* and *Treponema* spp. and 1.4% had findings of all three bacteria. The combinations of *D. nodosus*, *F. necrophorum* and *Treponema* spp. (44.4%) or *D. nodosus* and *Treponema* spp. (33.3%) were the most common findings in the 9 lambs with footrot score 2, whereas the most common finding in the 75 lambs with footrot score 1 was *Treponema* spp. (73.3%) and the combination of *D. nodosus* and *Treponema* spp. (13.3%). In the 428 lambs with footrot score 0, only 9.1% had findings of more than one bacterium, with *F. necrophorum* and *Treponema* spp. as the most common combination (5.8%).

The results of the regression analysis showed a significantly higher RRR of finding the combination of *D. nodosus* and *Treponema* spp. compared with *Treponema* spp. alone in lambs with footrot score 1 (RRR = 5.70; 95% CI 2.31–14.06; P < 0.001) or score 2 (RRR = 47.08; 95% CI 7.13–310.84; P < 0.001) than in lambs with footrot score 0. Moreover, the RRR was significantly higher for finding the combination of *D. nodosus*, *F. necrophorum* and *Treponema* spp. compared with only *Treponema* spp. in lambs with footrot score 2 (RRR = 230.37; 95% CI 29.86–1777.18; P < 0.001) than in lambs with footrot score 0. The RRR for finding the combination of *D. nodosus* and *Treponema* spp. was also significantly higher compared to finding no bacteria in lambs with footrot score 1 (RRR = 9.77; 95% CI 2.57–37.15; P = 0.001) than in lambs with footrot score 0. The RRR was significantly higher for finding the combination of *D. nodosus* and *Treponema* spp. compared with finding the combination *F. necrophorum* and *Treponema* spp. in lambs with footrot score 1 (RRR = 3.79; 95% CI 1.10–13.03; P = 0.035) than in lambs with footrot score 0. The RRR was significantly higher for finding the combination of *D. nodosus* and *Treponema* spp. compared with finding the only *Treponema* spp. in lambs with footrot score 2 (RRR = 8.25; 95% CI 1.22–55.85; P = 0.03) than in lambs with footrot score 1.

### Real-time PCR of single swab sample (CODD lesion)

The ESwab taken at the coronary band of the lamb assessed as CODD grade 1 tested positive for *Treponema* spp. and *F. necrophorum* subsp. *funduliforme* by real-time PCR, but negative for *F. necrophorum* subsp. *necrophorum* and *D. nodosus*.

### Geographical distribution

The geographical distribution of the nine lambs with footrot score 2 and the 75 lambs with footrot score 1 is shown in Table 4. Seven of the 311 lambs from southern Sweden (2.3%; 95% CI 0.9–4.6%), one lamb out of 155 from Gotland (0.6%; 95% CI 0.02–3.5%), and one lamb out of 46 from northern Sweden (2.2%; 95% CI 0.05–11.5%) had footrot score 2 (Table 4). There was no statistically significant difference in prevalence between the three regions, although there was a tendency for the RRR of finding lambs with footrot score 2, compared with finding lambs with footrot score 1, to be higher in southern Sweden compared with Gotland (RRR = 2.03; 95% CI 0.11–4.18; P = 0.06). Thirty-two of the 311 lambs from southern Sweden (10.3%; 95% CI 7.1–14.2%), 35 lambs out of 155 from Gotland (22.6%; 95% CI 16.3–30.0%) and eight lambs out of 46 from northern Sweden (17.4%; 95% CI 7.8–31.4%) had footrot score 1. The RRR of finding lambs with footrot score 1, compared with finding lambs with footrot score 0, was significantly higher on Gotland than in southern Sweden (RRR = 2.50; 95% CI 1.48–4.23; P = 0.001). No other significant associations were found between footrot score and region.

**Table 4:** Geographical distribution of footrot scores in Swedish slaughter lambs

Geographical region	Footrot score 0 (%)	Footrot score 1 (%)	Footrot score 2 (%)	Number of lambs, total
Southern Sweden^a^	272 (87.5)	32 (10.3)	7 (2.3)	311
Northern Sweden^b^	37 (80.4)	8 (17.4)	1 (2.2)	46
Gotland^c^	119 (76.8)	35 (22.6)	1 (0.6)	155

^a^Slaughterhouses located in Alunda, Hörby, Kalmar, Ljungskile and Skara

^b^Slaughterhouses located in Luleå and Vikarbyn

^c^Slaughterhouse located in Visby

*D. nodosus* was detected in 28 of the 311 lambs from southern Sweden (9.0%; 95% CI 6.1–12.7%), two out of 155 from Gotland (1.3%; 95% CI 0.2–4.6%) and one out of 46 from northern Sweden (2.2%; 95% CI 0.06–11.5%) (Table 5). The odds ratio (OR) of finding *D. nodosus* was significantly higher for lambs from southern Sweden (OR = 7.57; 95% CI 1.78–32.2; P = 0.006) than for lambs from Gotland. No other significant associations were found between detection of *D. nodosus* and geographical region.

**Table 5:** Distribution of *Dichelobacter nodosus*, *Fusobacterium necrophorum* and *Treponema* spp. in slaughter lambs from different geographical regions of Sweden

Geographical region	Number of lambs with *D. nodosus* (%)	Number of lambs with *F. necrophorum* subsp. *necrophorum* (%)	Number of lambs with *F. necrophorum* subsp. *funduliforme* (%)	Number of lambs with *Treponema* spp. (%)	Number of lambs, total
Southern Sweden^a^	28 (9.0)	2 (0.6)	25 (8.0)	271 (87.1)	311
Northern Sweden^b^	1 (2.2)	0	2 (4.3)	46 (100)	46
Gotland^c^	2 (1.3)	0	10 (6.5)	147 (94.8)	155

^a^Slaughterhouses located in Alunda, Hörby, Kalmar, Ljungskile and Skara

^b^Slaughterhouses located in Luleå and Vikarbyn

^c^Slaughterhouse located in Visby

The two samples from lambs with footrot score 2 that tested positive for *F. necrophorum* subsp. *necrophorum* were both from southern Sweden (Table 5). No significant associations were found between detections of *F. necrophorum*, *F. necrophorum* subsp. *necrophorum* or *F. necrophorum* subsp. *funduliforme* and geographical region.

*Treponema* spp. was found in 271 of the 311 lambs from southern Sweden (87.1%; 95% CI 82.9–90.6%), 147 out of 155 from Gotland (94.8%; 95% CI 90.1–97.7%) and 46 out of 46 from northern Sweden (100%; 95% CI 92.3–100.0%) (Table 5). The OR of finding *Treponema* spp. was significantly higher for lambs from Gotland (OR = 2.71; 95% CI 1.34–5.95; P = 0.01) than for lambs from southern Sweden. No other significant associations were seen between findings of *Treponema* spp. and region; observations from northern Sweden were omitted since region predicted the presence of *Treponema* spp. perfectly in that case (i.e. 100% of the lambs from northern Sweden were positive).

## Discussion

This study has shown that the current prevalence of footrot in Swedish slaughter lambs is 1.8%, which is significantly lower than in the previous survey (2009) [18]. No statistically significant difference in the prevalence of footrot was found between the three geographical regions (northern Sweden, southern Sweden and Gotland). In the previous survey, a significantly higher prevalence of footrot was found in northern vs. southern Sweden, but this was unexpected, since most flocks with footrot were found in southern Sweden [18]. More samples from northern Sweden would probably have been required in both studies in order to obtain more reliable regional comparisons. We still chose to follow the design of the previous survey to make the best possible comparison of national footrot prevalence over time and to increase the chance of finding CODD, a rare disease in Sweden. The two cases of CODD diagnosed so far have both been in southern Sweden [24]. A single lamb (0.2%) was found with a CODD lesion (grade 1) in this study. However, grade 1 lesions have been shown to resolve on their own and can be caused by factors other than CODD, such as trauma [26].

Several measures against footrot have been taken in Sweden since 2009, which could explain the reduction in prevalence from 5.8% to 1.8%. The most obvious measure is the control programme and, although only a minority (4.2%) of Swedish flocks participate in the programme, almost all top breeding flocks are included. A footrot-free status is mandatory at three of the four Swedish pedigree auctions and is strongly recommended for all trading of breeding animals in order to reduce spread within the country [36]. Footrot has also received more general attention in Sweden since the previous prevalence study, which may have led to increased awareness among sheep farmers, veterinarians and others who come into contact with sheep. However, it is unlikely that the decline in prevalence can be attributed to more lame animals being sent for slaughter in the previous prevalence study compared to the present, given that the transport of lame animals has been banned in Sweden for many years. There are also no reports from the authorities that slaughter lambs are being retained by sheep farmers due to problems with footrot; this would have been noticed as an animal welfare issue and moreover, contact with a veterinarian is required in Sweden for antibiotics to be prescribed. Within the control programme itself, the number of cases of footrot has decreased from 20 in 2009 to five in 2020 [27]. Hoof health and infection prevention in sheep flocks have received further attention in the past 2 years since the first case of CODD was diagnosed in Sweden [24]. These targeted measures may be some of the reasons why the prevalence of footrot disease has decreased.

The prevalence of *D. nodosus* in Swedish slaughter lambs (6.1%) was determined for the first time in 2020. The proportion of asymptomatic carriers was shown to be twice the number of lambs with footrot. Even though all *D. nodosus*-positive flocks reported to the Swedish Board of Agriculture in 2019 were located in southern Sweden [37], *D. nodosus* was detected in all three regions investigated in this study. As expected, *D. nodosus* was most common in lambs with footrot score 2 (77.8%). However, detection of *D. nodosus* was significantly more common in lambs with score 1 than in those with score 0, and it may be possible that lambs with score 1 and positive results for *D. nodosus* were in early lesion progression and would have eventually developed footrot. The prevalence of *D. nodosus* and footrot observed in this study was lower than that reported previously for other countries [38, 39]. However, it is difficult to make direct comparisons between different countries as data collection methods and study design often differ, as well as the clinical assessment and the definitions of footrot used. All *D. nodosus* detected in this study was benign, which is consistent with the results from previous studies conducted in Sweden [16, 40, 41]. This differs from the situation in other countries such as the United Kingdom, Switzerland and Germany, where virulent strains were more frequently found than benign strains [4, 20, 38, 39].

The prevalence of *F. necrophorum* was 7.6%; this bacterium was more commonly found in lambs with footrot than in healthy lambs (footrot score < 2), which is consistent with findings in a previous Swedish field study [16]. The occurrence of *F. necrophorum* in sheep with footrot has been reported in several countries [4, 5, 42], but few prevalence studies on random sample sets have been conducted. This may be because *F. necrophorum* has long been considered ubiquitous in soil, but Clifton et al. [7] recently showed that *F. necrophorum* is actually rarely found in soils and that few animals excrete it in their faeces [7]. The subspecies *funduliforme*, which is considered less virulent than subspecies *necrophorum* [43], was most prevalent in Swedish slaughter lambs. This differs from findings in Maboni et al. [4], where the majority of the *F. necrophorum-*positive samples were of subspecies *necrophorum,* but more severe footrot lesions were included in that study and *F. necrophorum* has been suggested to aggravate damage to the feet [5].

*Treponema* spp. was detected in the majority of the slaughter lambs (90.6%) and there was no significant association between footrot score and the presence of *Treponema* spp. Similarly, in a previous field study *Treponema* spp. was found in 18 of 20 sheep flocks [16]. Duncan et al. [25] also found no association between *Treponema* spp. and feet with footrot and CODD lesions at a family level *(Spirochetaceae*). The real-time PCR used in this study and the previous Swedish studies [16, 35] detected the entire *Treponema* genus and did not distinguish between commensal and pathogenic species. The presence of *Treponema* may need to be investigated at species level to determine whether there is an association with diseased feet. Certain species of *Treponema*, such as *T. phagedenis*, *T. medium* and *T. pedis*, have previously been shown to be associated with CODD [23, 26]. However, a significant association of *Treponema* at a genus level and footrot was found in this study in lambs with findings of *D. nodosus* and *F. necrophorum* in combination with *Treponema,* which is in line with the study by Staton et al. [26]. In lambs with footrot in this study, *D. nodosus*, *F. necrophorum* and *Treponema* spp. or *D. nodosus* and *Treponema* spp. were the most common findings.

There was good compliance between the pathological and real-time PCR findings, although *D. nodosus* was detected in a lower proportion of lambs with footrot than in the previous prevalence study [18] (77.8% compared with 96.6%). Possible explanations are that the samples were pooled in this study and that the transport time was longer, which may have affected bacterial survival. Pooling of samples in a similar way but in groups of five has previously shown a slight decrease in analytical sensitivity [41]. Real-time PCR does not require viable bacteria, however, non-intact bacterial cells and free DNA may be lost during DNA preparation when centrifugation is used to concentrate samples. The low prevalence of *D. nodosus* and *F. necrophorum* accurately represents the situation of few lambs having footrot or CODD, especially since all *D. nodosus* found was benign and the majority of *F. necrophorum* was of the lesser virulent subspecies, *funduliforme.* The high prevalence of *Treponema* spp. needs to be investigated in more detail, preferably at a species level.

## Conclusions

The prevalence of footrot in Swedish slaughter lambs has been significantly reduced, from 5.8 to 1.8%, in the past 11 years. This decrease is positive for animal health and production in the sheep industry. It also indicates that preventive measures such as the national footrot control programme and elimination of footrot from affected flocks have had an effect on the prevalence of the disease. A single lamb (0.2%) was found to have a CODD lesion (grade 1). In Sweden, benign strains of *D. nodosus* seem to be the most common. Neither *D. nodosus* nor *F. necrophorum* were widespread among Swedish slaughter lambs, but both were more commonly found in lambs with footrot. *Treponema* spp. was very commonly found in lambs with and without footrot, but there is a lack of information on the individual *Treponema* species present in slaughter lambs and their potential pathogenicity.

## Data Availability

The datasets used and/or analysed in this study are available from the corresponding author on reasonable request.
